# A few genetic variants go a long way in differentiating *Penstemon* species

**DOI:** 10.1371/journal.pbio.3002322

**Published:** 2023-09-29

**Authors:** Yaniv Brandvain, Shelley Sianta

**Affiliations:** Department of Plant and Microbial Biology, University of Minnesota, Saint Paul, Minnesota, United States of America

## Abstract

Hybridizing species are usually maintained by genome-wide selection against introgression or by selection on a few “genomic islands”. This Primer explores the implications of a study which finds a new pattern – 60 SNPs spread across the genome differentiate Penstemon species with different pollinators.

## Teaser

The integrity of hybridizing species is usually maintained by genome-wide selection or by selection on a few genomic regions. A study published in *PLOS Biology* finds a different pattern—60 SNPs spread across the genome differentiate a *Penstemon* species pair.

The past two decades of research on speciation genomics have shown that a simple bifurcating tree—the major metaphor presented to describe the process of speciation—is a biological exception rather than the rule. Speciation genomic analyses have revealed that post-divergence gene flow is common and have tended to find one of two genomic patterns of introgression, each of which involve heterogeneity in interspecific divergence across the genome. One commonly reported pattern is a negative relationship between recombination rate and introgression, and this pattern is interpreted as genome-wide selection against minor parent ancestry [1]. The other is a largely homogenized genome with the exception of a few genomic regions (or “islands”) of high divergence [2], inferred to be caused by pervasive gene flow accompanied by strong selection in the few regions that govern species identity. The field of speciation genomics has a long interest in identifying and (over) interpreting “islands of divergence”—and much care is needed in interpreting these patterns, as heterogeneity in some measures of differentiation are expected, even without gene flow. Bona fide genomic islands are predicted to consist of numerous tightly linked loci (often captured in an inversion), such that the homogenizing effects of gene flow are less likely to break up the loci underlying key adaptations that differentiate the two species [3]. A study in this issue of *PLoS Biology* by Wessinger and colleagues [4] fits neither of these patterns particularly well, and therefore, further complicates and illuminates our understanding of the process of speciation and resulting patterns of divergence in the genome. This study stands out in the narrowness and dispersion of regions of divergence (21 genomic regions spread across 8 chromosomes) and their proximity to loci known to underlie key adaptive differences between species.

**Fig 1:**
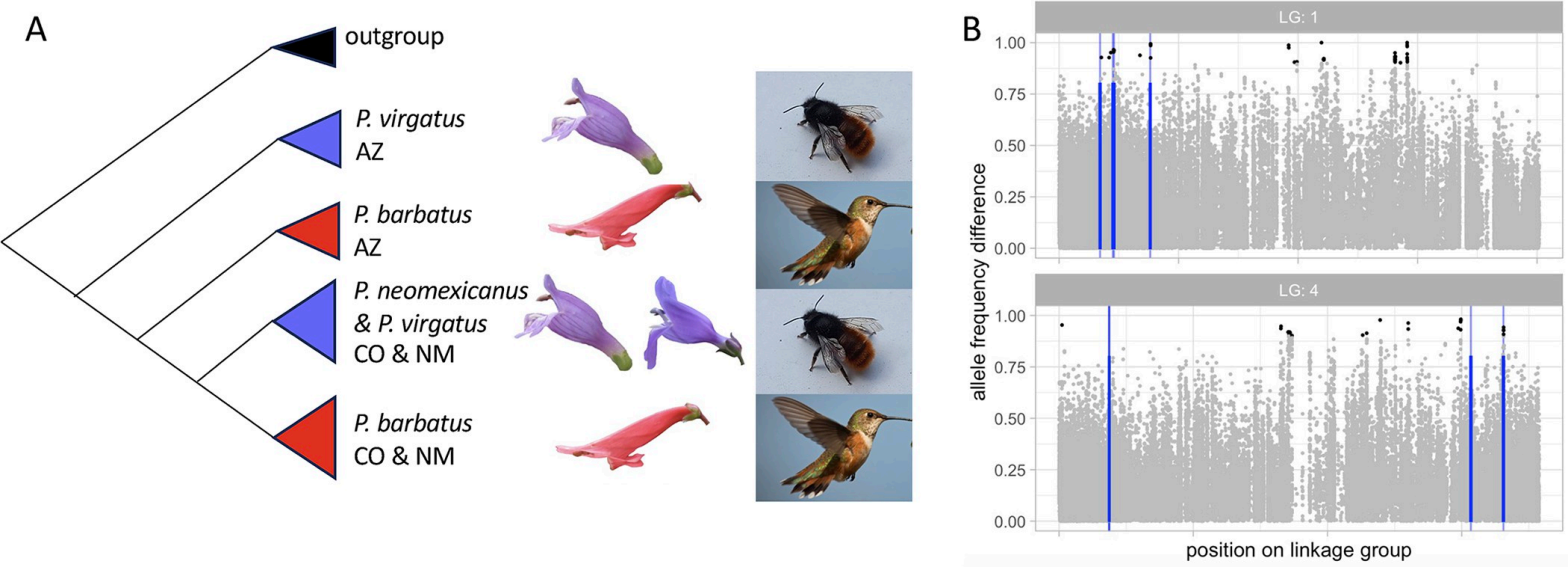
Genomics of divergence between bee-pollinated and hummingbird-pollinated *Penstemon*. (A) The genome-wide phylogenetic tree follows geography (US state abbreviated after species name), rather than pollinator syndrome—with blue and red designating bee-pollinated and hummingbird-pollinated taxa, respectively. (B) Example of patterns of genomic differentiation on linkage groups 1 and 4. Allele frequency differences are plotted on the y-axis, with extreme allele frequency differences highlighted in black. Blue lines denote the best estimate of the location of loci associated with quantitative traits underlying the pollination syndrome. Photo credits: Flowers are from Wessinger and colleagues [4]. Bees are made available under CC1.0 from Wikimedia Commons (https://commons.wikimedia.org/wiki/File:Bumblebee_on_a_white_windows_sill_in_Kiev.jpg). Birds are made available on Wikimedia Commons (https://commons.wikimedia.org/wiki/File:USFWS_ribes_sanguineum_(26123508822).jpg) by Peter Pearsall/USFWS.

The study of complex and phenotypically obvious adaptations (e.g., benthic versus limnetic fish, annual versus perennial plants, divergent wing morphology) that distinguish otherwise similar populations have long served as a model to understand the process of speciation [5]. Shifts in pollination syndromes, i.e., a complex suite of traits that are selected for by a particular type of pollinator, are a particularly strong example. Speciation associated with a transition between pollinator syndromes in *Penstemon* species is the result of repeated shifts from bee to hummingbird pollination, with the shifts in floral traits forming the basis of taxonomic species delineation. Wessinger and colleagues [4] focus on a species complex with a shift from bee to hummingbird pollination, where the species co-occur in the southern Rocky Mountains and sky islands of Arizona and New Mexico. With low coverage whole-genome resequencing, Wessinger and colleagues [4] discovered that the hummingbird-adapted *P*. *barbatus* is not monophyletic, but rather appears as two distinct subclades within the broader diversity of the bee-pollinated *P*. *virgatus* and *P*. *neomexicanus* (Fig 1A). Specifically, *P*. *barbatus* populations from Colorado and New Mexico are more closely related to *P*. *virgatus* and *P*. *neomexicanus* from these geographic regions than they are to *P*. *barbatus* populations from Arizona.

Despite this genome-wide pattern of low interspecific divergence relative to intraspecific diversity, Wessinger and colleagues [4] found 60 SNPs strongly associated with species identity. These SNPs are located in 21 genomic regions sprinkled across 8 chromosomes. Moreover, 8 of the SNPs associated with species identity were very close (i.e., <100 kb) to the best estimate of the position of three quantitative trait loci (QTL) defining the pollination syndromes (petal color, nectar volume, and flower width). This pattern would be surprising under a null model in which the position of these QTL were unrelated to the position of loci with extreme allele frequency differences between the species. Another two regions of exceptional allele frequency differentiation between species fell within 2 Mbp of the best estimate of the position of QTL for stamen length and petal angle. Fig 1B provides visual examples of both patterns of allele frequency differentiation and the best estimate of the positions of QTL underlying shifts in pollinator syndrome across two linkage groups. Finer mapping-resolution and larger mapping populations (and/or association mapping) will allow for a stronger evaluation of the hypothesis that the additional differentiation observed in this species-pair—i.e., SNPs with very different frequencies in the species, but that are far from the QTL explaining a majority of variation in their associated traits—is due to polygenic selection on loci of modest effect. Ultimately, this will allow us to understand how complex polygenic adaptation can be maintained across many small genomic regions despite porous species boundaries.

As de novo genome assemblies and large-scale resequencing efforts continue to spread to non-model species, solving the major problem of connecting patterns of divergence across the genome to the underlying process of speciation [6] is becoming more urgent. The exciting and unusual pattern of genomic divergence in *P*. *barbatus*/*P*. *virgatus*—with dozens of SNPs associated with species identity spread across the genome and colocalizing with loci underlying species differences despite isolation by distance both within and between species—is potentially consistent with numerous alternative evolutionary histories (e.g., the maintenance of complex adaptations in the face of gene flow, selection on standing variation, or the adaptive introgression of this adaptation across space). The authors are careful not to overinterpret the observed pattern, but suggest that the patterns observed are best explained by occasional local gene flow between species (despite the paucity of hybrids observed in the field) with strong assortative mating and divergent selection maintaining divergence in floral syndromes. While these patterns and analysis are exciting, it is not the only such case—regions associated with coloration differences between two subspecies are spread across multiple chromosomes in hybridizing subspecies of the northern flicker, *Colaptes auratus* [7]. It is currently unclear if this pattern is rarely identified because it is in fact rare, or if the bias against identifying small regions of divergence—which was overcome by careful SNP-by-SNP analysis in this paper—is responsible for the perceived rarity of this pattern of divergence.

The future of speciation genomics research is quite exciting. Combining genetic mapping and genome scans (like those of Wessinger and colleagues [4] and that of Hench and colleagues [8] which found exceptional inter-chromosomal linkage disequilibrium and extreme allele frequency differentiation near loci underlying difference in pigment and vision between species of hamlet fish that are reproductively isolated by visually based assortative mating) provides insights into the loci, and their genetic architecture, that mediate species differences. Moreover, efficient simulation of realistic models of divergence and selection in SLiM [9] and with machine learning approaches [10] will be key in our ongoing efforts to better understand the diverse processes of speciation by giving us a better picture of what processes in the past have resulted in the genomic patterns of differentiation we observe today. The field of speciation genomics is now well positioned to both connect patterns of genomic and genetic differentiation, to the evolutionary processes that generated these patterns. Such studies will inform our understanding of the process of speciation and will allow for the estimation of key parameters that are predicted to shape the genomic architecture of divergence between hybridizing populations (e.g., [3]).
